# A Study of Sedentary Behaviour in the Older Finnish Twin Cohort: A Cross Sectional Analysis

**DOI:** 10.1155/2014/209140

**Published:** 2014-04-15

**Authors:** Maarit Piirtola, Jaakko Kaprio, Annina Ropponen

**Affiliations:** ^1^Department of Public Health, Hjelt Institute, University of Helsinki, P.O. Box 41 (Mannerheimintie 172), 00014 Helsinki, Finland; ^2^UKK Institute, Centre for Health Promotion Research, Tampere, Finland; ^3^Department of Mental Health and Substance Abuse Services, National Institute for Health and Welfare, P.O. Box 30 (Mannerheimintie 166), 00300 Helsinki, Finland; ^4^Institute for Molecular Medicine (FIMM), University of Helsinki, P.O. Box 20 (Tukholmankatu 8), 00014 Helsinki, Finland; ^5^Finnish Institute of Occupational Health, Topeliuksenkatu 41 aA, 00250 Helsinki, Finland

## Abstract

The aim of the study was to investigate the effects of age, sex, and body mass index (BMI) on total sitting time among the Finnish twin cohort. Also, heritability and environmental factors were analysed. The final sample included 6713 twin individuals 53–67 years of age (46% men). Among them there were 1940 complete twin pairs (732 monozygotic [MZ] and 1208 dizygotic [DZ] twin pairs). Sedentary behaviour was queried with a self-reported questionnaire with multiple-choice questions about sitting time at different domains. The mean total sitting time per day was 6 hours 41 minutes (standard deviation: 2 hours 41 minutes). The total sitting time was less in women than in men (*P* = 0.002). Older age was associated with less total sitting time (*P* < 0.001). Those with higher body mass index had higher total sitting time in age and sex adjusted analysis (*P* < 0.001). MZ pairs were more similar for sitting time than DZ pairs, with initial estimates of heritability for the total sitting time of 35%.The influence of shared environmental factors was negligible (1%), while most (64%) of the variation could be ascribed to unique environmental factors, the latter including measurement error.

## 1. Introduction

Sedentary behaviour, measured as sitting time, is one of the major global public health concerns [1, 2]. A high amount of sitting is independently associated with overweight [3, 4] and cardiometabolic risk [4]. In addition, a high amount of sitting time increases all-cause and cardiovascular disease-related mortality independent of whether a person is meeting physical activity guidelines [5–7]. Thus, actions to investigate the backgrounds and genetics of sedentary behaviour need to be studied further in order to implement effective preventive actions [8].

Sedentary behaviour has been defined in various ways [9]. It has been suggested that sedentary behaviour can be a paradigm of its own, distinctive to that of moderate- to vigorous-intensity physical activity, with independent effects on health [2]. Thus, sedentary behaviour is not simply the absence of moderate- to vigorous-intensity physical activity [2] or even the presence of light physical activity [9]. Recently one of the globally accepted suggestions for the use of the term sedentary behaviour had been given by the Sedentary Behaviour Research Network, including the definition of sedentary behaviour “as any waking behaviour characterized by an energy expenditure ≤1.5 metabolic equivalents (METs) while in sitting or reclining posture” [10]. The Network also suggests using the term inactive in describing “those who are performing insufficient amount of moderate- to vigorous-intensity physical activity (MVPA) (i.e., not meeting specific physical activity guidelines)” [10].

In addition to variation in definitions, sedentary behaviour can also be measured in various ways. Even though there is a recommendation that sedentary behaviour should be monitored by incorporation of both self-reported and device-based measures [11], no consensus exists for a golden rule of method to measure sedentary behaviour [11].

In the observational research, sedentary behaviour has been investigated with self-report questions about sitting time during different sedentary activities such as watching TV or using the computer and sitting at work or in vehicles [12, 13]. Especially in large population-based studies, those involving thousands of participants, the self-reported sitting time is a useful method despite its inaccuracy of the total amount of physical activity and the potential recall bias [11].

Understanding why some people are physically active and others inactive or behaving in a sedentary way is important in planning public health interventions [14]. It has been shown that age, health status, self-efficacy, and motivation in addition to social and physical environmental factors are associated with physical activity levels [14]. In two recently published Finnish population-based studies using self-administered questionnaires, the mean sitting times per day in men and women 25 to 60 years of age varied between 5.5 to 6.9 hours and 5.2 to 6.4 hours, respectively [15, 16]. The total sedentary times in these Finnish cohorts [15, 16] were less than reported in a large multiethnic cohort study with over 130,000 older subjects where the average daily sitting time was 8.0 hours in men and 8.2 in women [7]. It has been reported that differences in sitting times are based on ethnicity, age, educational level, and body mass index (BMI) [17].

Another potential factor related to physical activity, inactivity, and sedentary behaviour is family background and genetic predisposition [18]. It has been shown that when exercise participation is determined as 60 minutes/week at a minimum intensity of 4 METs, interindividual genetic differences accounted for 48–71% of variance in both sexes [19]. A recently published review presented a variance for genetics that was widely spread, as heritability estimates ranged from 0 to 85% for physical activity and from 25 to 60% for physical inactivity depending on definitions of physical activity levels, population, age, and other differences between studies [18]. Among adolescents, genetics has explained 72% to 85% of variance in exercise participation determined as sedentary, moderate, or vigorous exercise by METs [20]. The contribution of genetic factors to variation in sedentary behaviour frequency per week among 12–20 year-old boys was 35% to 48% and 19% to 34% among same aged girls [21]. In addition, the influence of shared environmental factors decreased along age in adolescence [21]. In another study, the role of genetics in MVPA was 59% among adolescents and 12% in young adults [22]. In adolescents, genetics explained 34% of the variance of sedentary time (per week), whereas shared environmental (household) explained 10% and unique environmental factors 56% of the variation [22]. The influence of genetics in sedentary behaviour in young adults was 28% of variance suggesting a somewhat increasing impact of unique environmental factors along age. In older adult twins, genetics explained 31% of the time spent in sedentary behaviour (≤1.5 METs) whereas it was larger (47%) for MVPA [23]. The effect of unique environment (i.e., the exposures and experiences mainly related to adulthood) was 52–55% of variance [23]. It is possible that genetic influences are different in inactivity and sedentary behaviours compared to physical activity but there is an inconsistency between previously published results [18, 23].

In this paper, the focus was to describe the latest data collection of physical activity and sedentary behaviour in the Finnish twin cohort [24, 25]. The main aim was to investigate the relative contribution of genetics and environmental factors of sedentary behaviour in 53 to 67 year-old men and women from the Finnish twin cohort study. In addition, the effects of age, sex, and body mass index (BMI) on total sitting time were investigated.

## 2. Materials and Methods

### 2.1. Participants

The population-based data of the older Finnish twin cohort was available for 16,269 same-sex twin pairs [26]. The extensive longitudinal data includes four waves of surveys (baseline in 1975 and three follow-up surveys in 1981, 1990, and 2011/12) [24–26]. During the fourth wave (October 2011 to June 2012) all twins born 1945–1957 (*n* = 11 738) originally identified to the cohort in 1974 and known to be alive in 2011 with an address in Finland were sent the questionnaire survey, either in Finnish or in Swedish. All subjects, except 13 individuals, had answered at least one of the earlier surveys (1975 and/or 1981 and/or 1990) along the follow-up. The vital status of the original cohort members was updated in 2011 from the national Finnish population register. All surviving twins received the survey irrespective of their cotwin's status. The data collection of the fourth wave is mainly described in the review article in 2013 [25]. The protocol was designed and performed according to the principles of the Helsinki Declaration and was approved by the Ethical Committee of the Helsinki University Central Hospital.

### 2.2. Methods

To maintain continuity the original questions (such as in 1975 and 1981) were used wherever possible, despite the development of better measurements for some topics. The questionnaire of the fourth wave included comprehensive questions about self-reported health, functional capacity, and lifestyle factors, described in more detail in the review published in 2013 [25].

#### 2.2.1. Physical Activity

All four surveys included questions about the quality and quantity of leisure-time physical activities: amount (per year), duration (per one session), frequency (per month), and intensity of leisure-time physical activity as well as daily time of commuting by physically active means (including walking, jogging, and cycling) to and from work (minutes per day) (see Appendix A). Also physical workload of the present or previous work was asked.

#### 2.2.2. Sedentary Behaviour

Sedentary behaviour has been queried in the fourth questionnaire with multiple-choice questions about sitting time during different activities (see Appendix B). The participants have answered how many hours, on average, they are sitting per day: (1) in office or similar places, (2) at home watching TV or videos, (3) at home at the computer, (4) in a vehicle, and (5) elsewhere. Each question had four alternatives: (a) less than an hour, (b) an hour–less than two hours, (c) two hours–less than four hours, and (d) four hours or more. We assumed the intensity of each of these sedentary activities to be no more than 1.5 METs [9]. Total daily sitting time was the sum of the midpoints of the specific sitting categories, using 30 minutes for “less than an hour,” 90 minutes for “an hour–less than two hours,” 180 minutes for “two hours–less than four hours,” and 300 minutes for “four hours or more.” For those twin individuals (*n* = 4034) who were not working at the moment of the survey, sitting time at work was denoted as zero minutes.

### 2.3. Statistical Methods and Data Analysis

**Figure 1:**
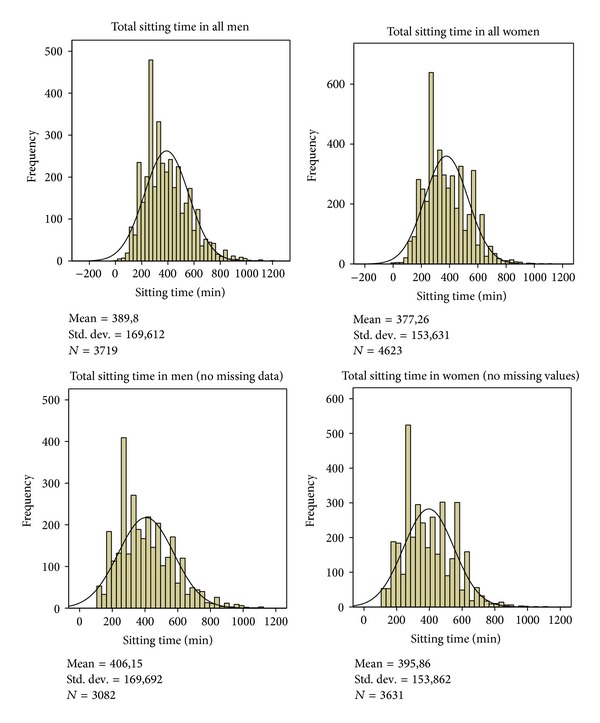
Distribution of total sitting time (minutes) by sex and data included. Total sum score includes all five domains: work, commuting, watching TV, computer use, and others. No missing data includes those with no missing data on any single sitting domain. For those not working, sitting time at work was denoted as zero minutes.

The total sitting time parameter has been developed based on five sitting activities by developing a summary variable (sitting time in hours and/or in minutes). Only those twin individuals reporting sitting times in all five sitting domains, including those not at work with zero sitting time on that item, were chosen for the final analyses. The normality of the sitting summary variable was tested with Kolmogorov-Smirnov and Shapiro-Wilk tests. The total sitting sum variable had the skewness value of 0.676 and kurtosis of 0.345 indicating that sitting time was not fully, normally distributed (*P* < 0.001) (Figure 1). However, tests of normality are extremely robust [27] and our relatively large sample size will result in a minor departure from normality being significant. Furthermore, the methods used in analyzing twin data are robust to minor deviation of normality. The original cohort had been a sample of twin pairs and that was taken into account, and robust standard errors were derived to obtain correct confidence intervals and *P* values [28]. The chi-squared test, Spearman correlation, and independent-sample* t*-test were used in the descriptive analyses. In the linear regression models, with 95% confidence intervals (95% CI), the effects of sex and age were analysed together. Age was used as a continuous parameter in the analyses.

Body mass index (BMI) was calculated by individual's weight and height (kg∗m^−2^). BMI values 20 or less as well as BMI 36 or more were combined to be the first and the last categories for descriptive purposes. The sex and age adjusted linear regression model with 95% confidence intervals was used in analysing the association between the total sitting time and BMI.

To investigate the heritability of physical activity, the phenotype was assumed to have an underlying, continuous liability. Heritability was analysed by calculating pairwise correlation coefficients by zygosity and sex and further comparing the results of monozygotic twins (MZ) to same-sex dizygotic twins (DZ). As MZ twins are genetically alike, that is, share the same genomic sequence, while DZ twins share on average 50% of the their segregating genes, increased similarity of MZ pairs versus DZ pairs is taken as evidence for the presence of genetic effects. In addition, Falconer's formula [29] was used to calculate the proportion of variance estimated by the ratio of MZ and DZ twin correlations explained by additive genetics (*h* ^2^ = 2(*r*_MZ_ − *r*_DZ_)), shared environmental factors (*c* ^2^ = 2*r*_DZ_ − *r*_MZ_), and unique environmental factors (*e* ^2^ = 1 − *h* ^2^ − *c* ^2^) based on MZ correlations being twice that or less compared to DZ correlations.

All analyses were performed with the Stata version 12 or the IBM SPSS version 21. In all analyses, significance was considered to be *P* < 0.05.

## 3. Results

The fourth questionnaire was returned by 8406 twin individuals (3750 men, 4656 women) resulting in a response rate of 72%. Complete data of sedentary behaviour (those answered in all five sitting domains) was available for 6713 participants (3082 men, 3631 women). The data covered 80% of those who returned the questionnaire.

There were more women (61% versus 54%, *P* = 0.009) in those having missing information at least in one sitting domain compared with those with information in all five domains. Those with any missing information were older (mean age 62 years versus 60 years, *P* = 0.000), not working full time (81% versus 41%, *P* = 0.000), and they were more likely obese, BMI > 30 (19.1% versus 16%, *P* = 0.009). There were no significant differences in the amount of leisure-time physical activity between those with or without missing information about their sitting times (*P* = 0.068).

The final analysis sample (*n* = 6713) comprised 310 complete monozygotic male pairs (MZM), 422 monozygotic female pairs (MZW), 527 dizygotic male pairs (DZM), and 681 dizygotic female pairs (together 1940 pairs). The average age of the twins was 60 years (range 53 to 67 years) in both sexes and their mean BMI was 26 (range 15 to 48). At the time of the survey, 59% of the twin individuals reported working full time. The physical workload of the present work was mainly sedentary work in 38% of the twins; 12% of twins had work which involved standing and walking but no other physical activity; 41% had work which in addition to standing and walking required lifting and carrying; and 8% did heavy physical work, whereas 1% of the twins reported a mixed combination of all these kinds of work loading conditions.

The mean sitting time per day was 6 hours 41 minutes (SD: 2 h 41 minutes) (Figure 1). In men, the mean total sitting time was 6 hours 46 min (SD: 2 h 50 minutes) and in women 6 hours 34 minutes (SD: 2 hours 34 minutes). One quarter of the twins reported sitting 4.5 hours or less per day, a half 4.5–6.5 hours a day, and 10% at least 10.5 hours a day. The sitting times during different sitting activities by sex are described in Table 1.

There were 6% of the individuals who reported not exercising any kind of physical activity during their leisure time. Of the sample, 22% reported a small amount of exercise and the rest 72% were exercising at least a moderate amount of exercise per year. The amount of leisure-time exercise by sex is described in Table 1. In the preliminary analyses, there was no evidence for differences in physical activity levels regarding the total daily sitting time (data not shown).

In the linear regression analyses, the total sitting time of women was less than sitting time of men (regression coefficients: −13.01 minutes [95% CI: −21.30, −4.83]) and increase of age decreased the sitting time (regression coefficients: −9.67 minutes per year [95% CI: −10.72, −8.63]). In the analyses of sitting time in different activities (Table 2), sex had no effect on the sitting time at work but sitting at work decreased with age. Both sexes had an equally long sitting time at home watching TV or videos, and age increased this activity. However, men had significantly longer sitting time at the computer at home as they did for sitting time in vehicles compared to women. Increase of age increased computer related sitting time but decreased the amount of vehicle-related sitting time. In other activities, both sexes were sitting an equal amount of time but age increased the sitting time.

**Figure 2:**
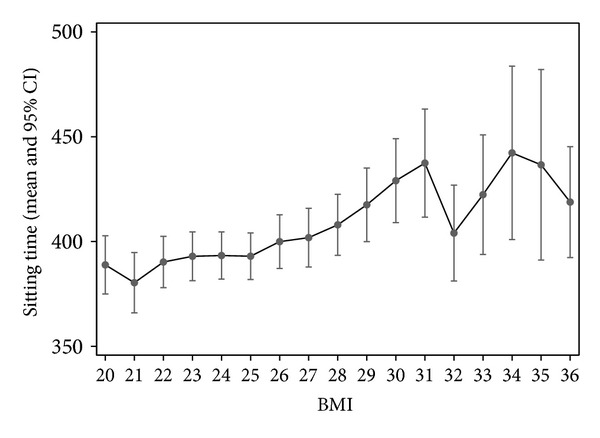
Association of BMI and regression model predicted mean of the total sitting time (minutes per day), with 95% confidence intervals. BMI values 20 or less as well as values 36 or more were combined to be the first and the last categories.

As an example of the sedentary behaviour risk factors, the association of body mass index with the total sitting time was analysed. The association of BMI and total time of sitting seemed to be linear in direction that those with higher BMI had also higher total sitting time (regression coefficients 2.78 minutes per BMI unit [95% CI: 1.77, 3.79], and correlation coefficient 0.064) (Figure 2).

In general, the pairwise correlations of MZ twins were double compared to correlation of DZ twins suggesting genetic influences on sedentary behaviour (Table 3). The correlation coefficients were similar for men and women. MZ pairs were more similar for sitting time than DZ pairs, with initial estimates of heritability for the total sitting time being 35%. The influence of shared environmental factors was negligible (1%), while most (64%) of the variation could be ascribed to unique environmental factors, the latter including measurement error.

## 4. Discussion

In this sample of 6713 twin individuals, 53–67 years of age, the total amount of sitting was on average 6 hours 41 minutes per day. In a Finnish population-based study of 30–45 years of age, the mean sedentary time (time spent viewing TV, using the computer, reading, listening to music/radio, and in other types of relaxation) was slightly less, a little over 5 hours, [16] but the time spent in vehicles was not inquired into. Another study, involving those of 25–64 years of age, has also shown similar estimates based on the mean self-reported sitting times (including sitting times at work and during leisure time, at home, while visiting friends, studying, and travelling) during a day: 6.9 hours (SD: 3.5) in men and 6.4 (SD: 3.3) hours in women [15].

In this study, the association between sitting time and age indicated that those of a younger age had higher total sitting time. The association of aging in sedentary behaviour and physical activity patterns is still poorly known [30]. Some evidence exists that younger adults are more active in moderate to vigorous physical activity than older adults [31] but knowledge is lacking on changes in the daily proportion of age-specific sedentary time. Among those with an average 79 years of age, sedentary behaviour explained 24% of the daily functions [30]. The changes in leisure-time activities after working age can only be speculated. In this study, 59% of the twin individuals reported working full time during the survey. It is possible that after working age the mean activity level may increase because of more active hobbies and a decreasing amount of sitting in vehicles to and from work, at least for a few years. This hypothesis needs to be studied further with long-term follow-up studies.

Our results also indicate that the increase in total sitting time is associated with increase of BMI. This is in line with other studies where a high amount of TV viewing time has been related to higher BMI and waist circumference [3, 16]. On the other hand, lower BMI has been related to a higher physical activity level in the aged [30].

There is evidence that genetics has at least a moderate influence on physical activity levels, and age seems to be a regulator of the activity heritability [21, 32, 33]. In adolescents (13–19 years of age), genetic factors explained 72–85% of the variance in exercise behaviour [20]. In another study, genetics of sedentary behaviour in 12-year-old boys was 35% and 19% in girls, whereas the proportion of variance explained by genetics increased to 48% in 20-year-old boys and to 34% in 20-year-old girls, respectively [21]. To the best of our knowledge, our study is among the first ones to study the relative contribution of heritability and environmental factors to sedentary behaviour, measured as total sitting time among older adults. In the present study, the influence of heritability for the total sitting time was 35%, and most (64%) of the variation could be ascribed to unique environmental factors. The role of heritability was of equal importance for women and men in this study. The proportion of heritability has been shown to be alike also in a study measuring daily activities with an accelerometer device [23]. However, the genetic component of physical inactivity has reported to be stronger in a comprehensive review [18] compared to our results. It has been reported that the heritability of physical activity decreases with age [33]. However, more sophisticated analyses need to be performed to confirm these results, also using relevant adjusting or stratified variables.

It seems that environmental factors play an important role in sitting time that may be related to adulthood choices or other factors unique to each individual such as occupation and leisure-time activities. The strong influence (50% to 72%) of unique environmental factors on physical activity has also been reported in other twin studies [22, 34]. These findings suggest that factors influencing our sedentary behaviour should be further elucidated. If the adulthood choices or habits, but also built environments are really of importance for the sitting time, as our preliminary results suggest, there might be possibilities to target public health campaigns to increase the public awareness of sedentary behaviour and/or to target both individual and community-based interventions in order to minimize sedentary behaviour and increase more activity and health enhancing behaviour. Societal policies in urban planning, the work environment, accessing leisure-time facilities, and many others are probably of great importance in their impact on total sitting time. However, we can also explore the role of earlier life circumstances, personality factors, life events, and health status using the cohort data available to us.

One of the main strengths of this study is the large population-based twin data and a high participation rate. The twin study design enables analysing the genetic component of sitting time. Also the generalizability of this twin data should be good since earlier reports have shown that the twins do not differ from the general population in terms of several traits including behaviour [35] or morbidity and mortality [25]. Thus, our study gives new information and aspects in analysing both the prevalence of sedentary behaviour by sitting activity and the relative role of heritability in sitting time.

Recommendations exist that monitoring self-reported sedentary behaviour should include overall sitting time in various activities [11]. In this study, the questionnaire included questions of sitting time in several activities; at work, at home, during commuting, and in all other activities from which we calculated the total time of sitting. Hence, we would like to assume that we have captured well the sitting time during a day. Even though there are both validity and reliability problems in self-rating methods reporting sedentary behaviour [36], there is also evidence that those who are reporting more sedentary behaviour in the self-rating questionnaires are also behaving more sedentary in the objectively measured studies [13]. Previous studies have used predominantly only TV viewing time or TV viewing alongside related “screen time” activities such as computer and video-based time as an indicator of sedentary behaviour [7, 11]. For example, long TV viewing time has been associated with overweight [3], mortality related to all-causes and cardiovascular diseases [7] as well as mental health [37] independent of many other risk factors or health behaviours. In addition, sitting most of the day has been shown to cause negative effects on insulin sensitivity and plasma lipids [38]. It is, however, noticeable that not all kinds of sitting are harmful to our health [37]. Further studies of the age-related sedentary behaviour heritability are needed.

## 5. Conclusion

The amount of sitting time decreases with increasing age but seems to increase along with BMI among older adults. Heritability seems to have a modest role in sitting time with no difference by sex.

## Figures and Tables

**Table 1:** Domain-based sitting times and the amount of leisure-time physical exercise by sex (*n* = 6713*).

Activities	Men (*n* = 3082)	Women (*n* = 3631)
	Mean time (SD)	Mean time (SD)
Sitting time		
At work**	2 h 52 min (1 h 53 min)	2 h 58 min (2 h 56 min)
At home watching TV or videos	2 h 25 min (1 h 12 min)	2 h 25 min (1 h 11 min)
At home at the computer	57 min (52 min)	50 min (43 min)
In a vehicle	59 min (59 min)	43 min (33 min)
Elsewhere	48 min (45 min)	48 min (45 min)
Sum of sitting time***	6 h 46 min (2 h 50 min)	6 h 36 min (2 h 34 min)

	*n* (%)	*n* (%)

Amount of leisure-time physical exercise****		
Practically none	252 (8)	150 (4)
A little	754 (25)	716 (20)
A moderate amount	1369 (45)	1722 (48)
Quite a lot or a great deal	695 (23)	1038 (29)

* Data from those twin individuals who had no missing values in any of the sedentary domains.

** Data from those twin individuals (*n* = 3970) who reported working full time at the moment of the survey.

***In calculating the total sum of sitting, sitting at office (work) was denoted as zero minutes for those twins (*n* = 2728) who reported not working at the moment of the survey (missing information in 15 individuals).

****Missing information in 17 individuals.

**Table 2:** Influence of sex and age on sitting time in five different sitting domains in the linear regression model (regression coefficients with 95% confidence intervals). Men were used as the reference sex.

Sitting domain	Coefficient	95% CI	*P* value
At work*			
Female sex	6.40	0.50, 12.30	0.034
Age per year	−11.92	−12.64, −11.20	0.000
At home watching TV or videos			
Female sex	1.10	−2.57, 4.76	0.558
Age	2.28	1.77, 2.79	0.000
At home at the computer			
Female sex	−6.70	−9.13, −4.26	0.000
Age	0.52	0.18, 0.87	0.003
In a vehicle			
Female sex	−16.86	−19.31, −14.41	0.000
Age	− 1.16	−1.46, − 0.86	0.000
Elsewhere			
Female sex	0.93	−1.29, 3.14	0.413
Age	0.74	0.44, 1.05	0.000

*For those twins who were not working at the moment of survey, sitting time at work was denoted as zero minutes (*n* = 2728).

**Table 3:** Pairwise correlations for sitting time by zygosity.

	Number of pairs	Correlation coefficient
MZ total	732	0.364
DZ total	1208	0.188
Male MZ	310	0.355
Female MZ	422	0.372
Male DZ	527	0.211
Female DZ	681	0.166

MZ: monozygotic; DZ: dizygotic.

## Appendices

### A. Questions about Physical Exercise

What is your daily time of commuting by physically active means (including walking, jogging, cycling, and/or cross-country skiing) to and from work in total?

- less than 15 minutes
- 15 minutes–less than half an hour
- half an hour–less than an hour
- an hour or more
- I am not presently at work

Leisure-time physical exercise (exercise that does not occur at work or while commuting to and from work). Here are five alternatives that describe the amount of your leisure-time exercise. Which one applies best to you when considering the amount of exercise you get during the year as a whole?

- practically none
- a little
- a moderate amount
- quite a lot
- a great deal

How long does one session of your leisure-time physical exercise last an average?

- less than 15 minutes
- 15 minutes–less than half an hour
- half an hour–less than an hour
- an hour to less than two hours
- over two hours

Presently how many times per month do you engage in physical exercise during your leisure time?

- less than once a month
- 1-2 times a month
- 3–5 times a month
- 6–10 times a month
- 11–19 times a month
- more than 20 times a month

Is your leisure-time physical exercise about as intensive on average as

- walking
- alternatively walking and jogging
- jogging (light run)
- running

### B. Question about Sitting Time (Sedentary Behaviour)

How many hours per day do you sit on average?

1. in office or similar places (e.g., during a working day):
  - less than an hour
  - an hour–less than two hours
  - two hours–less than four hours
  - four hours or more
2. at home watching TV or videos:
  - less than an hour
  - an hour–less than two hours
  - two hours–less than four hours
  - four hours or more
3. at home at the computer:
  - less than an hour
  - an hour–less than two hours
  - two hours–less than four hours
  - four hours or more
4. in a vehicle:
  - less than an hour
  - an hour–less than two hours
  - two hours–less than four hours
  - four hours or more
5. elsewhere:
  - less than an hour
  - an hour–less than two hours
  - two hours–less than four hours
  - four hours or more
